# Effect of miR-143-3p from Extracellular Vesicles of Porcine Uterine Luminal Fluid on Porcine Trophoblast Cells

**DOI:** 10.3390/ani12233402

**Published:** 2022-12-02

**Authors:** Yue Ding, Qun Hu, Jianyu Gan, Xupeng Zang, Ting Gu, Zhenfang Wu, Gengyuan Cai, Linjun Hong

**Affiliations:** 1 National Engineering Research Center for Breeding Swine Industry, College of Animal Science, South China Agricultural University, Guangzhou 510642, China; 2 Guangdong Provincial Key Laboratory of Agro-Animal Genomics and Molecular Breeding, South China Agricultural University, Guangzhou 510642, China; 3 Guangdong Laboratory for Lingnan Modern Agriculture, Guangzhou 510642, China

**Keywords:** pig, extracellular vesicles, ssc-miR-143-3p, porcine trophoblast cells

## Abstract

**Simple Summary:**

We found that miR-143-3p in extracellular vesicles of porcine uterine luminal fluid could be targeted and taken up by porcine trophoblast cells, with the effect of promoting cell proliferation and migration and affecting embryo implantation. We validated that miR-143-3p directly targets glycerol-3 phosphate dehydrogenase 2 (*GDP2*). Therefore, miR-143-3p and its target gene, *GPD2*, affect the function of porcine trophoblast cells and embryo implantation.

**Abstract:**

Extracellular vesicles (EVs) in uterine luminal fluid (ULF) can reportedly affect the proliferation and migration function of porcine trophoblast cells (PTr2 cells) by mediating the maternal–fetal exchange of information. miR-143-3p is considered a crucial miRNA in early pregnancy in mammals; however, little is currently known about how it regulates the function of PTr2 cells. This study aimed to investigate the effects of ssc-miR-143-3p in ULF-EVs on the function of PTr2 cells during porcine embryo implantation. The uptake of ULF-EVs by PTr2 cells was confirmed, which significantly increased the expression of ssc-miR-143-3p. Ssc-miR-143-3p was found to facilitate the proliferation and migration of PTr2 cells in the CCK-8, EdU and wound-closure assays, while the opposite findings were observed after the knockdown of ssc-miR-143-3p. Bioinformatics analysis and the luciferase reporter assay showed that glycerol-3 phosphate dehydrogenase 2 (*GDP2*) was directly targeted by miR-143-3p. Inhibition of miR-143-3p was validated in mice to inhibit embryo implantation. In summary, ssc-miR-143-3p in ULF-EVs affects the proliferation and migration of PTr2 cells by mediating *GPD2*, thereby affecting embryo implantation.

## 1. Introduction

Embryo survival rate is one of the major factors determining the fertility of sows. During the whole porcine pregnancy process, only approximately 60% of embryos eventually survive, and the remaining 20–30% experience death during the early and middle stages of gestation, which results in a serious waste of embryo resources and limited reproductive performance of sows [1,2,3]. Meishan pigs are currently recognized as one of the most productive pigs in the world, with significantly more piglets than the Yorkshire pig at the same ovulation rate [4,5]. Compared to Yorkshire pigs, Meishan pigs have a larger uterine capacity, which enables them to preserve more embryos throughout pregnancy [6]. Therefore, it is essential to improve porcine productivity by harnessing the excellent traits of the Meishan pig, which include low embryo mortality and high litter size.

Maternal–fetal exchange of information is reportedly crucial for embryo implantation. During implantation, the trophoblast cells in the blastocyst create tissue and physiological connections with the endometrial epithelium, which stabilize the development of the blastocyst in the uterus [7,8]. The critical phase in pigs is day 12 of gestation (gd12), whereby the embryonic morphology changes dramatically from tubular to filamentous, accompanied by the secretion of large amounts of estrogen and interleukins; estrogen levels peak on gd12 and can be harnessed to validate pregnancy to facilitate embryo implantation [9,10,11]. Given that the porcine embryo floats in the uterine luminal fluid (ULF) before implantation, the ULF serves as a necessary route of maternal–fetal interaction, and extracellular vesicles (EVs) in the ULF participate in information transmission [12]. EVs are wrapped in multiple miRNAs, mRNAs, active proteins and other substances, which are crucial for regulating intercellular communication. EVs are found in various body fluids, including serum, emulsion, urine, follicular fluid, seminal plasma and uterine luminal fluid [13,14,15,16,17,18,19]. Although miRNAs in ULF-EVs are essential for regulating maternal–fetal communication during the preimplantation phase, the specific mechanisms remain unclear.

MicroRNA (miRNA) is an endogenous non-coding RNA that consists of 20–23 nucleotides and inhibits the function of mRNA by binding to the 3’UTR fragment of mRNA [20,21,22]. miRNAs are widespread in animal reproductive organs and regulate various biological processes, including fertilization, embryo development, embryo implantation and maternal–fetal interaction [23]. miR-143 is a pivotal miRNA in early mammalian pregnancy and affects endometrial tolerance, embryo implantation and other reproduction-related events [24,25,26,27]. Additionally, human endometrial lesions are affected by miR-143-3p [28]. Cellular functions such as cell proliferation, migration and apoptosis are active in cancer cells, while embryonic cells behave highly similarly to cancer cells during embryo implantation [29,30]. An increasing body of evidence suggests that miR-143-3p is critical in cellular functions such as cell proliferation and migration [31,32]. Although the effects of miR-143-3p are reportedly critical in the mammalian reproductive system, the mechanism of ssc-miR-143-3p in ULF-EVs at the preimplantation stage for embryo implantation and maternal–fetal interaction remains obscure.

In this study, we aimed to investigate the regulation of porcine trophoblast cells (PTr2 cells) function and embryo implantation by ssc-miR-143-3p in ULF-EVs. The uptake of ssc-miR-143-3p in EVs by PTr2 cells was explored by detecting the expression of ssc-miR-143-3p in PTr2 cells. Furthermore, we investigated the mechanism by which ssc-miR-143-3p affected PTr2 cell function and validated the effect of ssc-miR-143-3p on embryo implantation using the mice model. This study provides a reference for understanding the regulation mechanism of embryo implantation by miR-143-3p in ULF-EVs and provides clues for improving the fertility of pigs.

## 2. Materials and Methods

### 2.1. Collection of Samples

The experimental population consisted of Meishan and Yorkshire pigs of similar ages and genetic backgrounds; all the pigs were artificially fed and bred for multiple generations in the same feeding and housing management. Five pigs per group were treated for simultaneous estrus and artificially inseminated at the onset of estrus (day 0) and again 12 h later. The uterus was removed on day 12, and the uterus was flushed with PBS to obtain ULF. The presence of filamentous embryos was used to confirm successful pregnancy, and three pregnant pigs per group were selected for this study. Meanwhile, tissue samples of the heart, liver, spleen, lung, kidney, ovary and endometrium were harvested.

### 2.2. Cell Culture and Transfection

PTr2 cells were isolated from porcine filamentous embryos at gd12 and provided by Dr. Yin Yulong from the Institute of Subtropical Agriculture, Chinese Academy of Sciences, China. These PTr2 cells were cultured in DMEM/F12 (Gibco, Grand Island, NY, USA) supplemented with 0.1% insulin (YEASEN, Shanghai, China) and 10% fetal Bovine serum (Gibco, Grand Island, NY, USA) at 37 °C and 5% CO_2_. When approximately 70% cell confluence was reached, cells were transfected using the Lipofectamine 3000 Kit (Invitrogen, Waltham, MA, USA). The synthesis of miR-143-3p mimics and antagomiR-143-3p was performed by GenePharma (Suzhou, China).

### 2.3. EVs Isolation

The particles in ULF were separated by centrifugation at different speeds [33]. The supernatant was collected by centrifugation at 2000× *g* for 20 min to remove cells and blood and at 10,000× *g* for 30 min to remove cell debris, large granular vesicles and apoptotic vesicles. Subsequently, impurities were removed from the supernatant using a 0.22 μm filter, and the precipitate was collected by ultracentrifugation at 120,000× *g* for 2 h. The above centrifugation step was carried out at 4 °C. Then, 100 μL PBS (Gibco, Grand Island, NY, USA) were used to resuspend the EVs and stored at −80 °C.

### 2.4. Transmission Electron Microscope (TEM) Assay

The EVs resuspended with PBS were added dropwise to the carbon membrane grid for 2 min, and excess liquid was removed from one end of the grid by aspiration with filter paper. After staining with uranyl acetate, it was left overnight at room temperature. Photographs were taken using transmission electron microscopy (Talos F200S, FEI, Waltham, MA, USA).

### 2.5. Extracellular Vesicles Delivery Analysis and Confocal Microscopy

Labeling of EVs was carried out according to the PKH67 kit (Sigma-Aldrich, St. Louis, MO, USA) instructions. In brief, 100 μL EV resuspension or PBS were added to 1 mL of Dilution C, respectively, followed by 4 μL PKH67 incubation for 4 min. Subsequently, the sample was closed with 2 times the volume of 0.5% BSA and leveled with PBS, then centrifuged at 120,000× *g* for 2 h at 4 °C. The precipitate was PKH67-labeled EVs.

Cells were fixed with 4% paraformaldehyde (Shyuanye, Shanghai, China) after coincubation with PKH67-labeled EVs or PBS with PTr2 cells for 48 h; the PTr2 cells were stained with TRITC Phalloidin (YEASEN, Shanghai, China) followed by DAPI staining according to the instructions. Subsequently, photographs were taken after 48 h of co-incubation using confocal microscopy (LSM 800, Zeiss, Oberkochen, Germany).

### 2.6. Protein Extraction and Western Blot

RIPA Lysis Buffer (CWBIO, Beijing, China) was added to the samples for total protein extraction. Samples were lysed at 0 °C for 20 min, centrifuged at 14,000× *g* for 7 min at 4 °C, and the supernatant was collected. Subsequently, after adding the protein loading buffer, the supernatant was denatured at 100 °C for 10 min.

The denatured protein samples underwent electrophoresis on 10% SDS-PAGE gels (Servicebio, Wuhan, China) at a constant pressure of 120 V for 1 h, followed by 1.5 h of membrane transfer at a constant flow of 0.2A and then blocked with 5% skimmed milk (Sangon, Shanghai, China) for 2 h. Rabbit polyclonal antibodies, including anti-TSG101 (cat. no, 381538, ZENBIO, Chengdu, China, 1:1000), anti- CD9 (cat. no, D264336, BBI Life Sciences, Shanghai, China, 1:1000), anti-HSP70 (cat. no, 10995-1-AP, Proteintech, Wuhan, China, 1:1000), anti-Calnexin (cat. no, 10427-2-AP, Proteintech, Wuhan, China, 1:1000), anti-GM130 (cat. no, abs127362, absin, Shanghai, China, 1:1000), anti-GPD2 (cat. no, ab188585, abcam, Cambridge, MA, USA, 1:1000) and β-tubulin (cat. no, 10094-1-AP, Proteintech, Wuhan, China, 1:1000), were incubated with samples at 4 °C overnight. The following day, the membrane was covered with a secondary antibody and placed on a shaker and incubated for 1.5 h at room temperature. The luminescent solution was configured using the ECL Western blot kit (CWBIO, Taizhou, China) and exposed using the EC3 imaging system (UVP). The membrane was washed 5 times (5 min each time) in TBST buffer (Servicebio, Wuhan, China) before each step after the transfer.

### 2.7. Real-Time Quantitative PCR (RT-qPCR)

Extraction of EVs RNA: Extracellular vesicle RNA extraction was performed according to the miRNeasy Serum/Plasma Kit (Qiagen, Hilden, Germany) instructions. Extraction of cellular and tissue RNA: Total RNA was extracted using TRIzol (Invitrogen, Carlsbad, CA, USA) according to the manufacturer’s instructions.

miRNA was reverse transcribed using the Poly(A) tailing method according to the reverse transcription kit (TaKaRa, Kyoto, Japan). The forward primer design was based on the mature sequence of ssc-miR-143-3p in the miRBase (http://www.mirbase.org/; accessed on 10 June 2021) database by changing U to T; the reverse primer was a universal primer. Combining the miRBase database with the NCBI database (https://www.ncbi.nlm.nih.gov/; accessed on 10 June 2021), the forward primer and reverse primer for pri-mir-143 were designed around 200 bp before and after the pre-mir-143 sequence. U6 was used as an internal standard, and cel-miR-39 was an external standard. The relative expression levels were assessed by the 2^−ΔΔCt^ method. The primer sequence information is presented in Table S1.

### 2.8. Nanoparticle Tracking Analysis (NTA)

The number of particles of EVs suspended in PBS by different particle sizes was counted using a ZetaView instrument (Particle Metrix, Meerbusch, Germany) and plotted using GraphPad Prism 9.0.

### 2.9. CCK-8 Assay

Cell proliferation was detected at 12 h, 24 h, 48 h and 72 h after transfection with the CCK-8 kit (YEASON, Shanghai, China). In a 96-well cell plate, 10 μL CCK-8 were added to each well of culture medium and placed at 37 °C for 1–3 h. After that, the optical density (OD) at 450 nm was analyzed using a microplate reader (Bio-Rad, Hercules, CA, USA).

### 2.10. EdU Assay

The BeyoClick™ EdU-555 kit (Beyotime, Shanghai, China) manufacturer’s instructions were used as a basis to detect cell proliferation 48 h after transfection. An equal amount of EdU working solution was added to the cell culture medium and cultured for 2 h. The samples were then fixed with 4% paraformaldehyde (Shyuanye, Shanghai, China) and permeabilized with immunostaining permeation buffer containing Triton X-100 (Beyotime, Shanghai, China). The click reaction mixture was added to the wells and incubated for 30 min, and the nuclei were stained using DAPI (Servicebio, Wuhan, China). Finally, images were taken with a fluorescent microscope (Nikon, Tokyo, Japan). Six randomly selected fields of view in each cell well were used to determine the proportion of proliferating cells in each well.

### 2.11. Wound-Closure Assay

Cells were cultured in 6-well plates at the appropriate density, and a cell monolayer was obtained after approximately 36 h transfection. Then, the cells were wounded by scratching with a 200 μL pipette tip. After removing the dislodged cells, the cells were serum starved to enable migration for 12 h. The extent of wound healing was recorded and photographed with a microscope and analyzed for area size using Image J. The wound closure rate was assessed using the following formula: migration rate = (0 h wound area − 12 h wound area)/0 h wound area.

### 2.12. Dual-Luciferase Reporter Assay

Three tools, including miRanda (http://www.microrna.org; accessed on 2 September 2021), RNAhybrid (http://bibiserv.techfak.uni-bielefeld.de/rnahybrid/RNAhybrid; accessed on 2 September 2021) and TargetScan (http://www.targetscan.org/; accessed on 2 September 2021), were used to predict target genes. The fragment of the binding site of ssc-miR-143-3p to the *GPD2* 3′UTR was added to the Pmir-GLO vector to construct the wild-type plasmid (WT-*GPD2*-3′UTR), and then the mutation vector (WUT-*GPD2*-3′UTR) was designed based on the sequence of the wild-type vector.

The dual luciferase reporter vector and miR-143-3p mimic or negative control (NC) were added to PK-15 cells and co-incubated for 24 h. Cells were collected according to the instructions of the Dual Luciferase Kit (YEASON, Shanghai, China), and luciferase activity was measured by a microplate reader (Bio-Rad, Hercules, CA, USA).

### 2.13. Injection Test

In vivo studies in pigs are difficult and uneconomical, and miR-143-3p is conserved in pigs versus mice. Therefore, we established an in vivo model using mice of the same week age, similar body weight and the same feeding conditions (*n* = 3) [34,35]. The day of vaginal plug detection was considered day 1 of pregnancy, and uterine horn injection was conducted on day 3. The ovaries and uterine horns were found on both sides of the back in mice under general anesthesia. Equal volumes of antagomiR143-3p or inhibitor negative control (inhibitor NC) were injected into each side of the uterine horn. Mice were sacrificed on day 7 of pregnancy, and the uterus was harvested to record the number of embryos implanted.

### 2.14. Statistical Analysis

All experiments in this study consisted of three biological replicates. Results were analyzed and graphed using GraphPad Prism 9.0. Qualitative data were expressed as mean ± standard deviation (Mean ± SD), and the t-test was used to compare whether there was a statistical difference between the two groups. * *p* < 0.05 was considered statistically significant, ** *p* < 0.01 was considered a highly significant difference between groups and ns indicated that it was not significant.

## 3. Results

### 3.1. Separation and Identification of EVs

**Figure 1:**
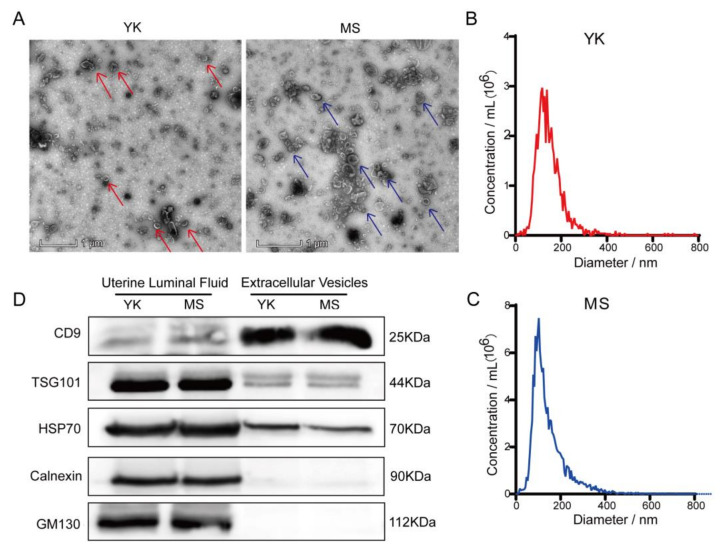
Characterization of extracellular vesicles (EVs) for identification. (**A**) Representative transmission electron microscope images are shown separately. The arrows point to extracellular vesicles. Scale bars = 1 μm. (**B**,**C**) Nanoparticle tracking analysis (NTA) detects the size of EVs, which are mainly distributed at 100 nm. (**D**) Western Blot detected marker proteins CD9, TSG101 and HSP70 in EVs; Calnexin and GM130 were used as negative controls. YK: Yorkshire pigs, MS: Meishan pigs.

Transmission electron microscopy showed that the EVs exhibited a membrane-like package with a central depression (Figure 1A). Nanoparticle tracking analysis showed that EVs had a particle size of approximately 100 nm (Figure 1B,C). Intriguingly, Western Blot results demonstrated that the surface marker proteins CD9, TSG101 and HSP70 of EVs were detected in ULF and EVs of Meishan and Yorkshire pigs on gd12, while the endoplasmic reticulum marker protein Calnexin and Golgi marker protein GM130 were only found in the ULF (Figure 1D; Figure S1, Supplementary Materials). Taken together, these results revealed that the purity of the extracted EVs was high enough for subsequent studies on miRNAs within EVs.

### 3.2. Expression Profile of ssc-miR-143-3p

**Figure 2:**
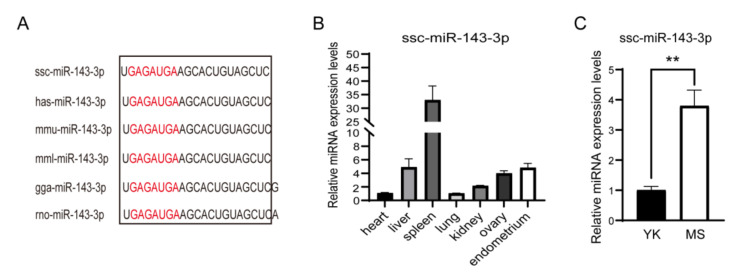
The expression characteristics of ssc-miR-143-3p. (**A**) Mature sequences of miR-143-3p showed that miR-143-3p was conserved across different species. (**B**) The expression characteristics of ssc-miR-143-3p in pig tissues. (**C**) Differential expression of ssc-miR-143-3p derived from extracellular vesicles in uterine luminal fluid (ULF-EVs) in Meishan and Yorkshire pigs. YK: Yorkshire pigs, MS: Meishan pigs. ** *p* < 0.01.

Conservativity analysis of miR-143-3p indicated no difference in the mature sequence of miR-143-3p across humans, pigs, rats, mice, monkeys and chickens (Figure 2A). In addition, the widespread expression of ssc-miR-143-3p was confirmed by RT-qPCR assay in various tissues of pigs (Figure 2B). Notably, the expression of ssc-miR-143-3p in ULF-EVs on gd12 was significantly higher in Meishan pigs than in Yorkshire pigs (Figure 2C). Therefore, ssc-miR-143-3p was chosen to study the effect of highly expressed miRNAs in ULF-EVs of Meishan pigs at gd12 on PTr2 cells during early pregnancy.

### 3.3. ssc-miR-143-3p Was Transported to PTr2 Cells by ULF-EVs

**Figure 3:**
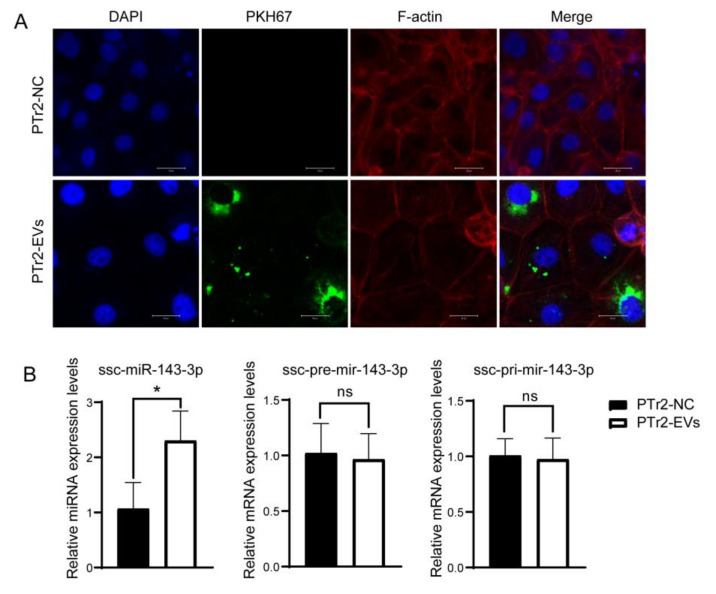
Ssc-miR-143-3p derived from extracellular vesicles in uterine luminal fluid (ULF-EVs) targeted transport to porcine trophoblast cells (PTr2 cells). (**A**) Internalization of ULF-EVs by PTr2 cells was observed by confocal microscopy. DAPI: Cytosolic dyes, PKH67: EVs dyes, F-actin: Cell membrane actin. Scale bars = 20 μm. (**B**) RT-qPCR detected significant upregulation of ssc-miR-143-3p expression in PTr2 cells after co-incubation with EVs. Negative control (NC), add an equal volume of PBS. * *p* < 0.05; ns—not significant.

To investigate whether PTr2 cells could endocytose ULF-EVs, the extracted EVs were labeled with PKH67 and co-incubated with PTr2 cells for 48 h. PTr2 cells exhibited uptake of EVs distributed around the nucleus under confocal fluorescence microscopy (Figure 3A). Notably, ssc-miR-143-3p was significantly increased in PTr2 cells after co-culture with ULF-EVs from Meishan pigs for 48 h, while ssc-pre-mir-143 and ssc-pri-mir-143 did not change significantly (Figure 3B). Taken together, the highly expressed ssc-miR-143-3p after EV treatment was transported into PTr2 cells by EVs rather than endogenously produced by the cells.

### 3.4. ssc-miR-143-3p Enhances the Function of PTr2 Cells

**Figure 4:**
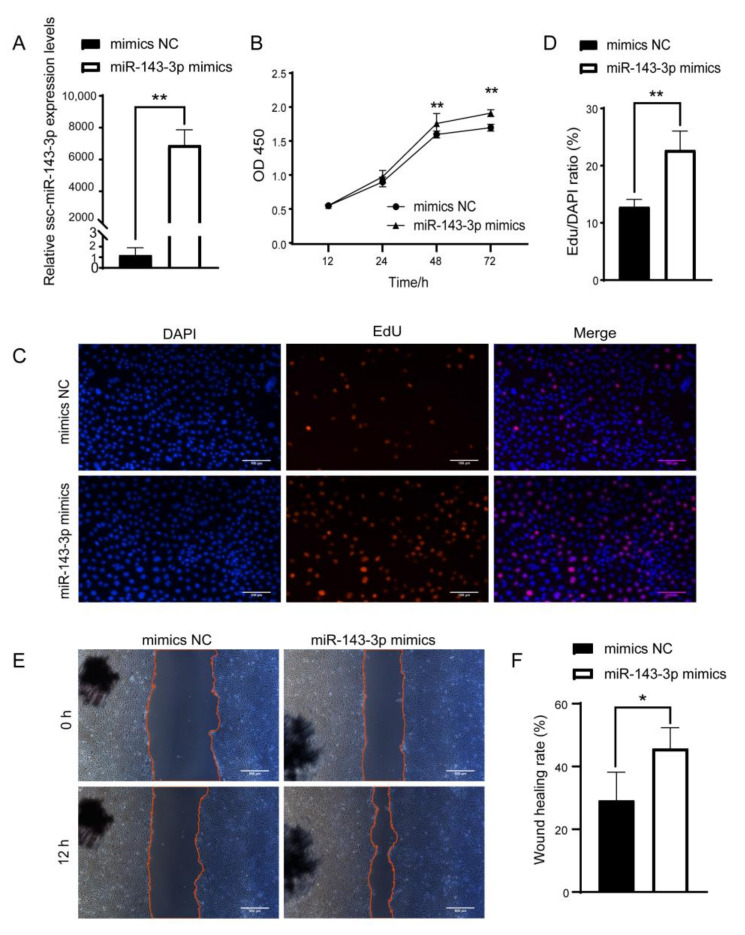
Upregulation of ssc-miR-143-3p promotes the proliferation and migration of porcine trophoblast cells (PTr2 cells). (**A**) The efficiency of ssc-miR-143-3p overexpression was examined by RT-qPCR. (**B**) Cell proliferation was detected by CCK-8, and ssc-miR-143-3p overexpression was found to promote the proliferation of PTr2 cells. (**C**,**D**) Altered cell proliferation after overexpression of ssc-miR-143-3p was detected by EdU. Scale bar = 100 μm. (**E**,**F**) Cell migration was detected by wound closure assay. Cells were photographed after 0 h and 12 h of cell damage, respectively. Scale bar = 500 μm. * *p*< 0.05; ** *p* < 0.01.

Although we established the uptake of ULF-EVs by PTr2 cells, the regulatory mechanism of ssc-miR-143-3p in EVs on the function of PTr2 cells remains unclear. To investigate the regulation of ssc-miR-143-3p on PTr2 cell function, PTr2 cells were transfected with miR-143-3p mimics to construct an overexpression model of ssc-miR-143-3p. The content of ssc-miR-143-3p in PTr2 cells increased dramatically, demonstrating high transfection efficiency (Figure 4A). CCK-8 assay demonstrated that higher levels of ssc-miR-143-3p significantly facilitated the proliferation of PTr2 cells (Figure 4B). Meanwhile, the EdU assay confirmed that ssc-miR-143-3p was beneficial in increasing the number of EdU-positive in PTr2 cells (Figure 4C,D). In addition, the wound-closure assay revealed that the upregulation of ssc-miR-143-3p significantly increased the speed of wound healing in PTr2 cells and facilitated cell migration (Figure 4E,F). Overall, high expression of ssc-miR-143-3p facilitated the function of PTr2 cells.

### 3.5. Inhibition of ssc-miR-143-3p Inhibited Proliferation and Migration of PTr2 Cells

**Figure 5:**
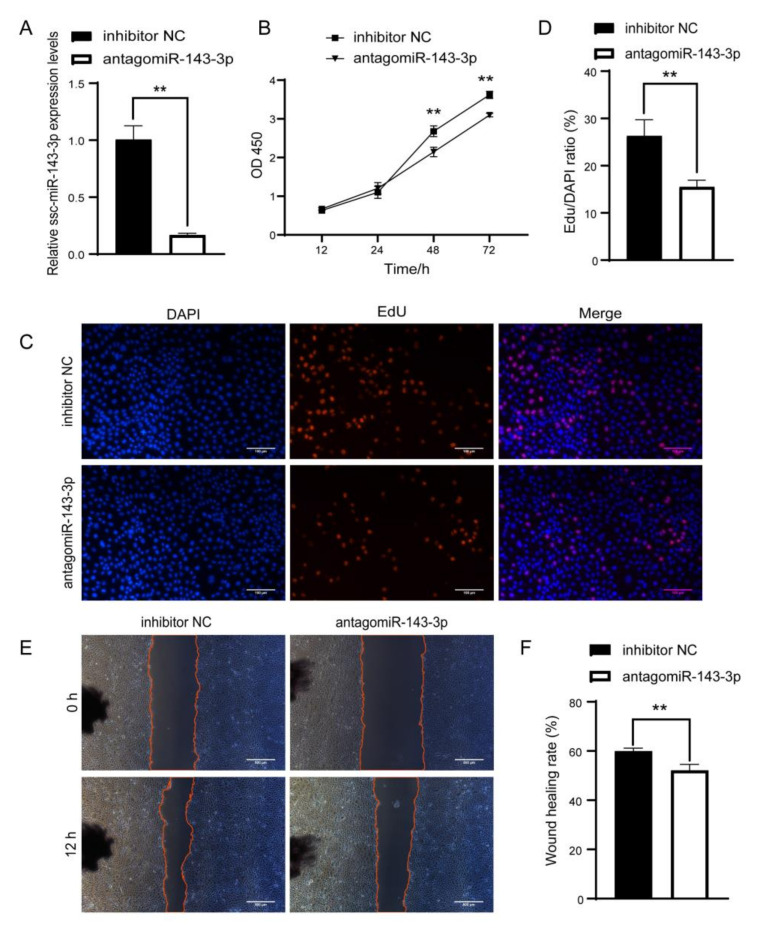
Inhibition of miR-143-3p inhibits the function of porcine trophoblast cells (PTr2 cells). (**A**) The efficiency of knocking down ssc-miR-143-3p was examined by RT-qPCR. (**B**) Cell proliferation was detected by CCK-8, and ssc-miR-143-3p overexpression was found to promote the proliferation of PTr2 cells. (**C**,**D**) Detection of EdU-positive PTr2 cells after transfection with antagomiR-143-3p. Scale bar = 100 μm. (**E**,**F**) Cell migration was detected by wound closure assay. Cells were photographed after 0 h and 12 h of cell damage, respectively. Scale bar = 500 μm. ** *p* < 0.01.

To further investigate the changes in PTr2 cell function after the knockdown of ssc-miR-143-3p, PTr2 cells were transfected with antagomiR-143-3p. Synthetic antagomiR-143-3p was introduced into the cells and dramatically downregulated its expression compared to the control (Figure 5A). CCK-8 analysis after transfection revealed that the knockdown of ssc-miR-143-3p significantly inhibited the proliferation of PTr2 cells (Figure 5B). Furthermore, the EdU assay showed that the percentage of cells in the proliferative phase was dramatically decreased after the knockdown of ssc-miR-143-3p (Figure 5C,D). Moreover, wound closure assays demonstrated that wound healing was dramatically slowed in PTr2 cells after inhibiting ssc-miR-143-3p (Figure 5E,F). In conclusion, ssc-miR-143-3p is indispensable in maintaining PTr2 cell functions, such as proliferation and migration.

### 3.6. miR-143-3p Targets Binding to 3′UTR of GPD2

**Figure 6:**
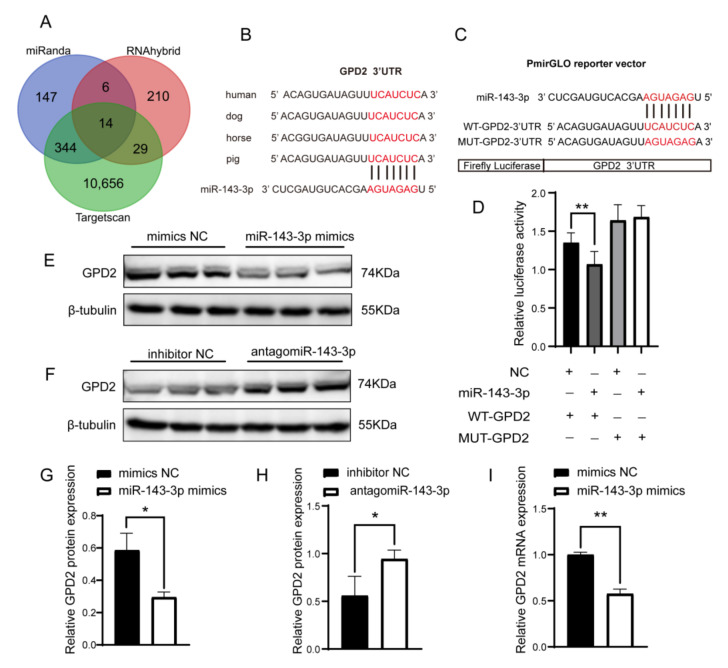
Targeting relationship between glycerol-3-3 phosphate dehydrogenase 2 (*GDP2*) and miR-143-3p. (**A**) In total, 14 target genes were predicted by miRanda, RNAhybrid and Targetscan databases. (**B**) The binding fragment of the 3’UTR of *GPD2* to the miR-143-3p seed region was highly conserved in different species. (**C**) Dual luciferase vector sequence design. (**D**) Dual fluorescein reporter gene validates direct targeting of miR-143-3p to *GPD2*. (**E**–**H**) Western Blot validates direct targeting of miR-143-3p to *GPD2*. (**I**) Validation of miR-143-3p for targeting and regulating *GPD2* by RT-qPCR. ** *p* < 0.01; * *p*< 0.05.

To investigate the regulatory mechanism of ssc-miR-143-3p in PTr2 cells, a total of 11,406 target genes were predicted using miRanda, RNAhybrid and Targetscan, of which 14 target genes were identified by the intersection of results of the three online tools (Figure 6A). Among these 14 target genes, glycerol-3-3 phosphate dehydrogenase 2 (*GDP2*) was reported playing an indelible role in regulating cell proliferation, migration, apoptosis and cell cycle [36]. Therefore, we selected *GPD2* for subsequent validation. The target sequence of the 3′UTR of the *GPD2* gene was highly conserved in mammals, including humans, dogs, horses and pigs (Figure 6B). Wild-type or mutant *GPD2*-3′UTR dual luciferase reporter plasmids were constructed, and dual luciferase reporter assays identified a significant decrease in dual luciferase activity after co-transfection of ssc-miR-143-3p with WT-*GPD2*-3′UTR, while co-transfection with MUT-*GPD2*-3′UTR showed no significant difference in dual luciferase activity (Figure 6C,D). Furthermore, Western Blot revealed a significant decrease in *GPD2* protein expression in PTr2 after upregulation of ssc-miR-143-3p, while knockdown of ssc-miR-143-3p exhibited the opposite results (Figure 6E–H; Figures S2 and S3, Supplementary Materials). In addition, RT-qPCR confirmed the inhibition of *GPD2* expression by ssc-miR-143-3p (Figure 6I). In conclusion, ssc-miR-143-3p could direct target *GPD2*.

### 3.7. Knockdown of miR-143-3p Led to Failure of Embryo Implantation in Mice

**Figure 7:**
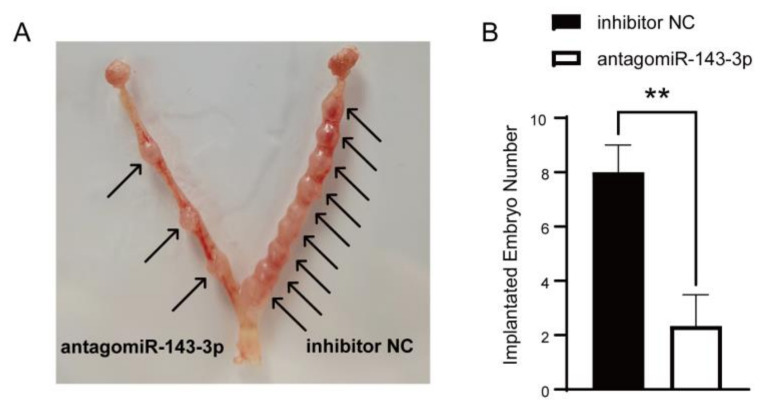
Inhibition of mouse embryo attachment by antagomiR-143-3p. (**A**,**B**) Comparison of the number of embryos implanted with antagomiR-143-3p or inhibitor negative control (inhibitor NC) injected in uterine horns. ** *p* < 0.01.

The effect of antagomiR-143-3p on embryo implantation was further demonstrated using mice models. AntagomiR-143-3p and inhibitor NC were injected into both uterine horns of mice on day 3 of pregnancy. We found that the number of embryos on the side of antagomiR-143-3p injection was significantly lower than the inhibitor NC injection side when the uterus was removed on day 7 of pregnancy (Figure 7A,B).

## 4. Discussion

Embryo implantation is an important event in the establishment of pregnancy during mammalian reproduction and significantly affects reproductive traits such as litter weight and size [37]. ULF-EVs are widely thought to mediate signal communication between the uterus and the blastocyst trophoblast during preimplantation [38]. CD9, TSG101 and HSP70 are surface marker proteins of EVs, Calnexin is a surface marker protein of the endoplasmic reticulum, which is only involved in EVs synthesis but not expressed in EVs, and GM130 is a Golgi surface marker protein [38,39]. Consistent with the literature, we found that EVs were positive for CD9, TSG101 and HSP70 and negative for Calnexin and GM130, indicating that the purity of EVs isolated in this study met experimental requirements.

ULF-EVs labeled by PKH67 have been reported to be observed in endometrial epithelial cells and PTr2 cells [33]. In addition, EVs derived from embryonic trophoblast cells were found to act on endometrial epithelial cells to promote embryo implantation [29]. In this study, EVs marked by PKH67 were co-incubated with PTr2 cells, and the uptake of ULF-EVs by PTr2 cells was observed. In summary, the ULF-EVs can be taken up and internalized by the embryo and the endometrium for maternal–fetal interaction. EVs were identified to be able to target their own wrapped miRNAs for transport into cells by detecting pre-miRNA and pri-miRNA expression [40,41]. In this study, EVs were co-incubated with PTr2 cells to quantify the expression of miRNA, pre-miRNA and pri-miRNA. The level of ssc-miR-143-3p was significantly higher in PTr2 cells treated with EVs compared with the control group, while ssc-pre-mir-143 and ssc-pri-mir-143 were not significantly changed. It was hypothesized that the high levels of ssc-miR-143-3p in PTr2 cells were transported into the cells by ULF-EVs rather than endogenously produced, which provides a foothold for subsequent investigation of the effect of miRNAs in EVs on PTr2 cell function.

Regulation of embryo implantation and embryo development by miRNAs in EVs has been a research hotspot in recent years, and various miRNAs have been associated with maternal–fetal interaction [42]. Studies have shown that the expression of miR-143 was significantly upregulated at the mouse embryo implantation site compared to the inter-implantation site and was involved in the modulation of physiological processes such as endometrial receptivity and embryo implantation in rats [24,25,26]. In this study, we found that the expression of ssc-miR-143-3p in ULF-EVs of Meishan pigs on gd12 was significantly increased than in Yorkshire pigs and speculated that the specific high expression of ssc-miR-143-3p in Meishan pigs might be relevant to embryo implantation and development. It has been established that PTr2 cells must have high proliferation and migration abilities for successful embryo implantation [43]. Current evidence suggests that decreased miR-218 in bovine endometrium-derived EVs inhibit trophectoderm cell migration and impair embryo development [44]. Furthermore, human endometrium-derived EVs were found to promote the adhesion function of HTR8 cells by regulating the FAK signaling pathway [45]. This study revealed that high expression of ssc-miR-143-3p significantly promoted the proliferation and migration ability of PTr2 cells, while downregulation of ssc-miR-143-3p inhibited PTr2 cell function. On gd12, the pig embryos elongated rapidly to a filamentous form and underwent drastic morphological changes within a short period [9]. Consequently, the promoting effect of ssc-miR-143-3p on the proliferation and migration ability of PTr2 cells may facilitate the rapid elongation of embryo morphology and implantation.

*GPD2* is a protein located in the inner mitochondrial membrane and is mainly associated with glycolysis, inflammatory immune response, cellular senescence and tumorigenesis [46]. Overwhelming evidence substantiates that *GPD2* is involved in regulating cell proliferation, migration and apoptosis [36]. In this study, three online tools, miRanda, RNAhybrid and Targetscan, were used to analyze *GPD2* as a possible target gene of ssc-miR-143-3p, and triple validation by a dual luciferase reporter gene, RT-qPCR, and Western blot further confirmed the existence of targeting relationship between them. In conclusion, it was suggested that ssc-miR-143-3p might affect embryo implantation by regulating *GPD2*. However, the specific mechanism of *GPD2* regulation on PTr2 cell function requires further investigation.

This experiment can further explore the regulatory role of *GPD2* on PTr2 cell function, and the specific regulatory pathway of miR-143-3p warrants further investigation.

## 5. Conclusions

In this study, we substantiated the uptake of ssc-miR-143-3p in ULF-EVs by PTr2 cells in Meishan pigs on gd12, which induced cell proliferation and migration by targeting *GPD2*, thereby regulating embryo implantation.

## Data Availability

All data are contained within the article or Supplementary Materials.

## Supplementary Materials

The following supporting information can be downloaded at: https://www.mdpi.com/article/10.3390/ani12233402/s1. Table S1: Primer information; Figure S1: Original western blot figures for Figure 1D; Figure S2: Original western blot figures for Figure 6E; Figure S3: Original western blot figures for Figure 6F.
