# Supplementary material for: Effect of miR-143-3p from Extracellular Vesicles of Porcine Uterine Luminal Fluid on Porcine Trophoblast Cells
Source: Animals (Basel). 2022 Dec 2;12(23):3402. doi: 10.3390/ani12233402 (PMC9736583; doi:10.3390/ani12233402)
Supplement: Supplementary file 1 [file animals-12-03402-s001.zip › animals-1998983-Original-Images for Blots-v3.pdf]

a

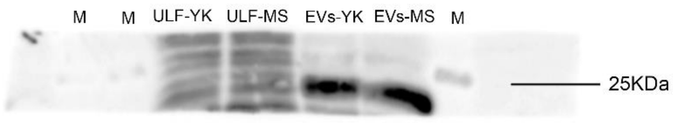
M
M
ULF-YK
ULF-MS
EVs-YK
EVs-MS
M
25KDa

b

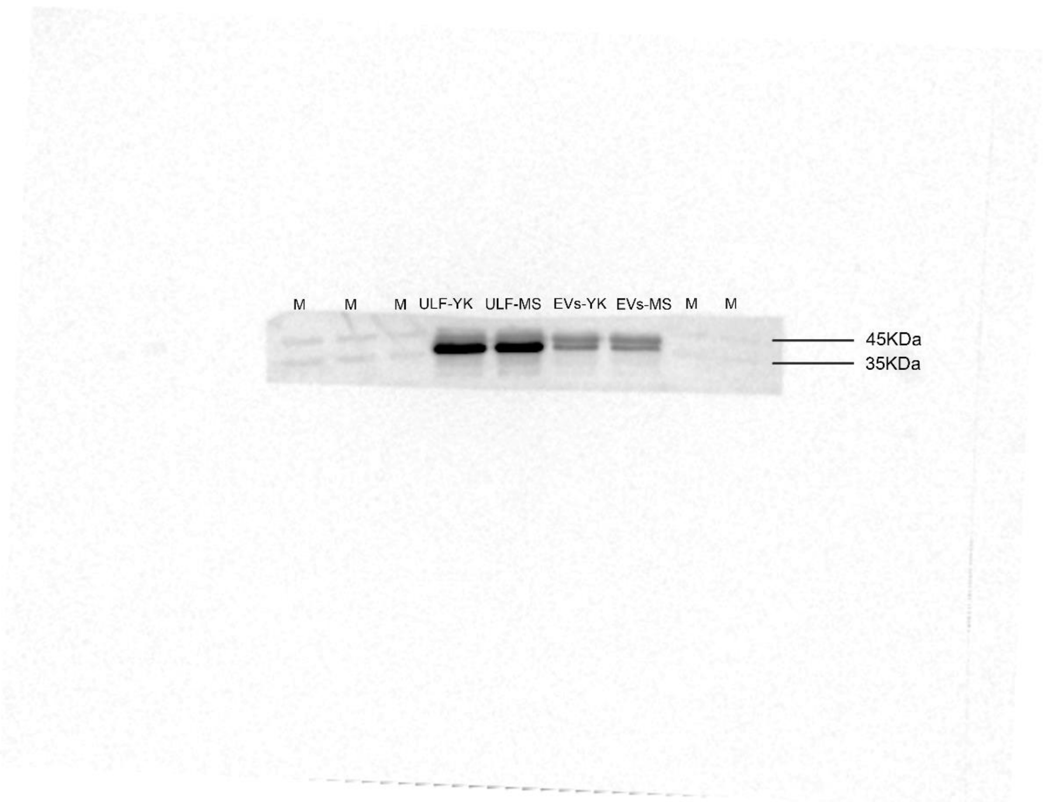
M
M
M
ULF-YK
ULF-MS
EVs-YK
EVs-MS
M
M
45KDa
35KDa

c

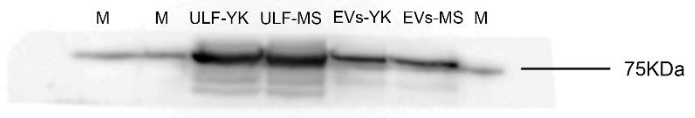
M
M
ULF-YK
ULF-MS
EVs-YK
EVs-MS
M
75KDa

d

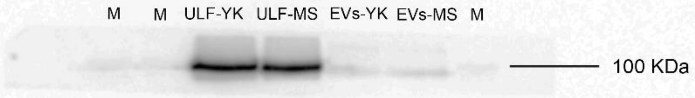
M
M
ULF-YK
ULF-MS
EVs-YK
EVs-MS
M
100 KDa

e

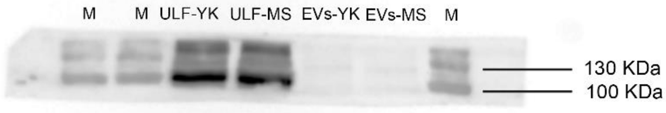

Figure S1. Original western blot figures for Figure 1D. (**a**) CD9, 25KDa. (**b**)TSG101, 44KDa. (**c**) HSP70, 70KDa. (**d**) Calnexin, 90KDa. (**e**) GM130, 112KDa. ULF: uterine luminal fluid, EVs: extracellular vesicles, YK: Yorkshire pigs, MS: Meishan pigs, M: marker.

a

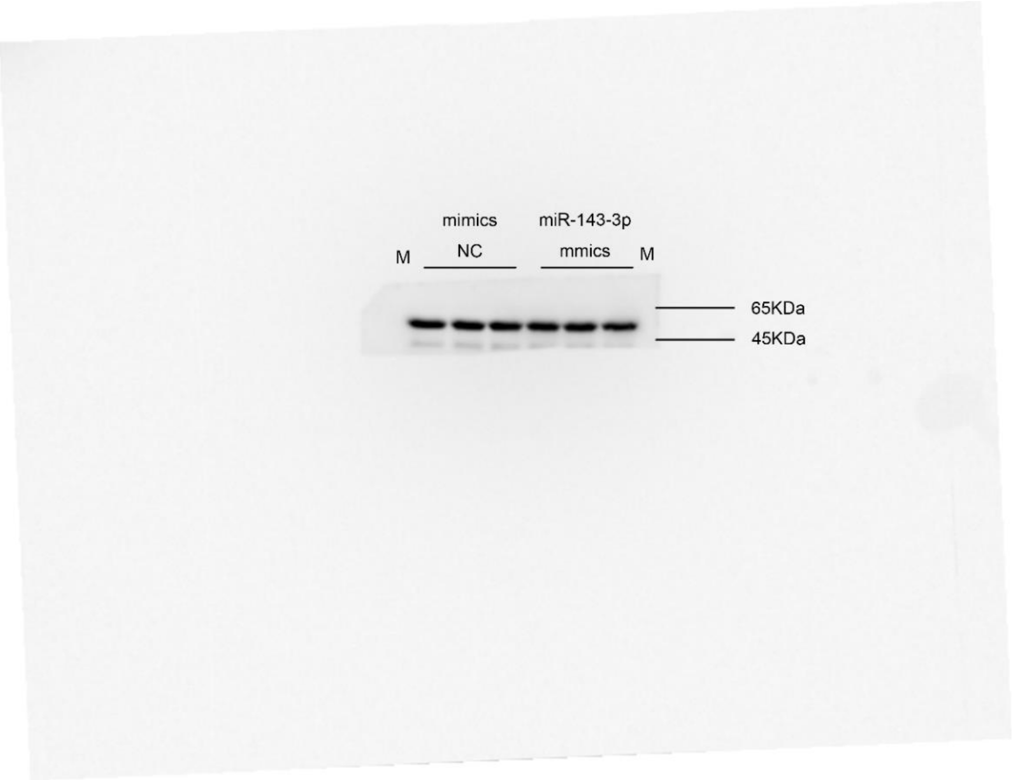

b

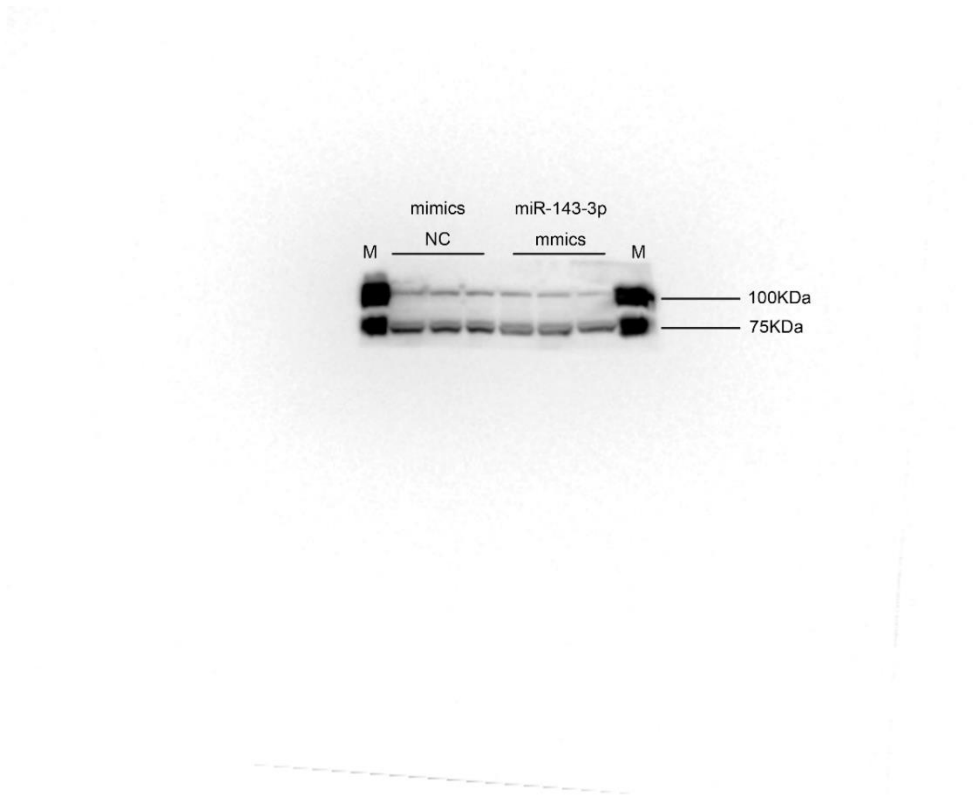

Figure S2. Original western blot figures for Figure 6E. (**a**) β-tubulin, 55KDa. (**b**)*GPD2*, 74KDa.

a

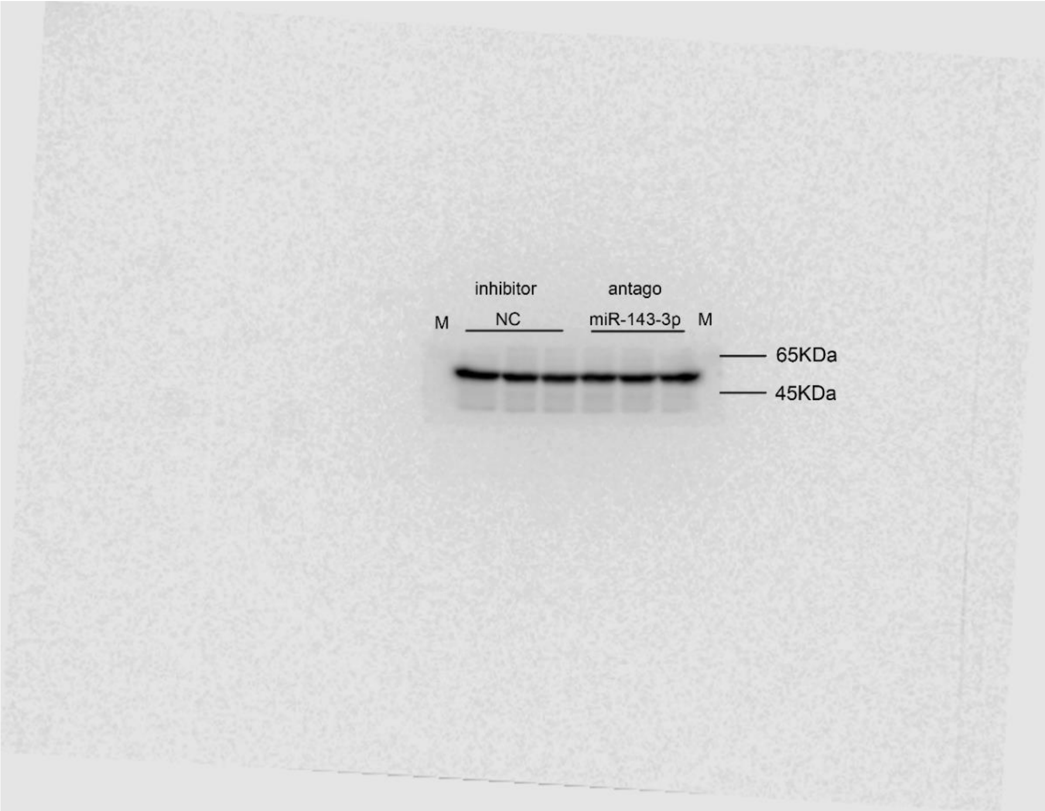

b

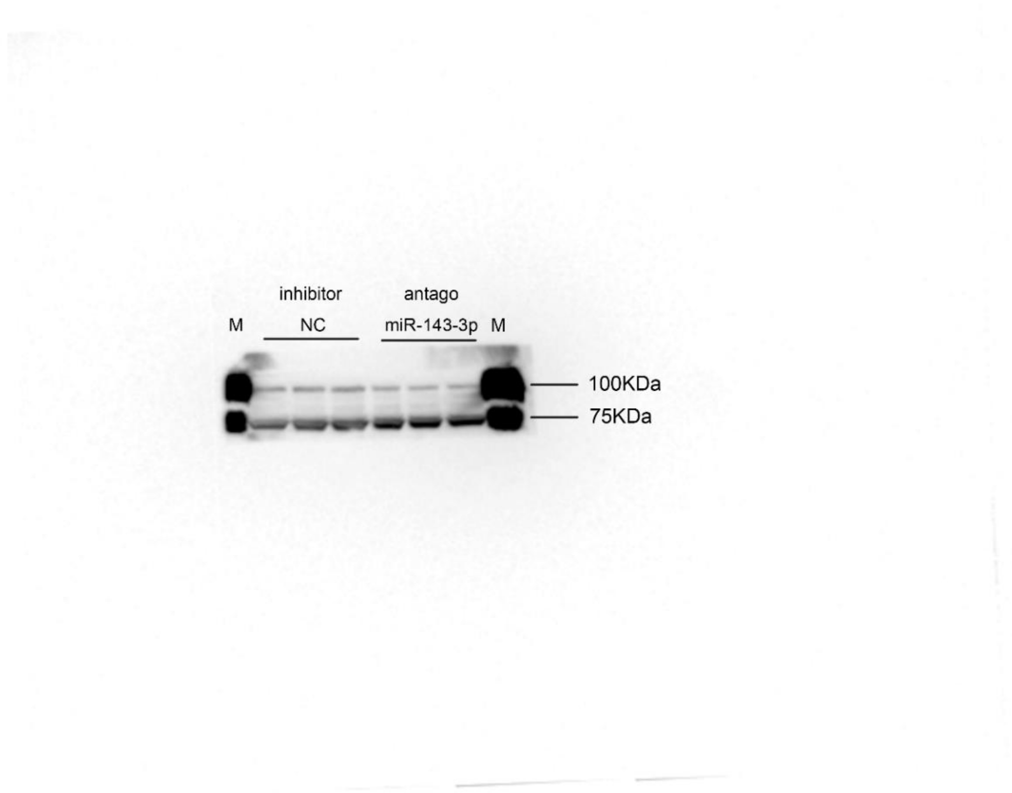

Figure S3. Original western blot figures for Figure 6F. (**a**) β-tubulin, 55KDa. (**b**)*GPD2*, 74KDa.
