# Supplementary material for: Effect of miR-143-3p from Extracellular Vesicles of Porcine Uterine Luminal Fluid on Porcine Trophoblast Cells
Source: Animals (Basel). 2022 Dec 2;12(23):3402. doi: 10.3390/ani12233402 (PMC9736583; doi:10.3390/ani12233402)
Supplement: Supplementary file 1 [file animals-12-03402-s001.zip › animals-1998983-Table S1.pdf]

Table S1 Primer information

Primer name	Sequence of primers
GPD2	F: CAAATGGATGAAAACACGCTT
	R: TCCACAACTTCGGTCTACAGG
β-actin	F: GTCATGGACTCTGGGGATGG
	R: TTCTCCTTGATGTCCCGCAC
ssc-pre-mir-143	F: AGTGCTGCATCTCTGGTCAGCTG
	R: GCAGCAGAGCAGCTTCTCCCTTC
ssc-pri-mir-143	F: AAGGGAAAGCAAGGTGGAGGC
	R: GCAGCAGAGCAGCTTCTCCCTTC
ssc-miR-143-3p	F: TGAGATGAAGCACTGTAGCTC
